# Surface Electromyography Data Analysis for Evaluation of Physical Exercise Habits between Athletes and Non-Athletes during Indoor Rowing

**DOI:** 10.3390/s24061964

**Published:** 2024-03-19

**Authors:** Tomasz Grzejszczak, Anna Roksela, Anna Poświata, Anna Siemianowicz, Agnieszka Kiełboń, Michał Mikulski

**Affiliations:** 1 Faculty of Automatic Control, Electronics and Computer Science, Silesian University of Technology, 44-100 Gliwice, Poland; 2 EGZOTech Sp. z o.o., 44-100 Gliwice, Poland; 3 Faculty of Science and Technology, University of Silesia, 75 Pułku Piechoty 1A, 41-500 Chorzów, Poland

**Keywords:** surface electromyography (sEMG), muscle activation measurement, physical exercise evaluation

## Abstract

In this paper, surface electromyography (sEMG) is used to gather the activation neural signal from muscles during an indoor rowing exercise. The exercise was performed by professional athletes and amateur non-athletes. The data acquisition and processing are described to obtain a set of parameters: number of cycles, average cycle time, cycle time standard deviation, fatigue time, muscle activation time, and muscle energy. These parameters are used to draw conclusions on common non-athletes’ mistakes during exercise for better training advice and a way of statistically distinguishing an athlete from a non-athlete.

## 1. Introduction

Nowadays, one can notice an increased willingness of people to take care of their personal physical development and practice amateur sports. Additionally, amateurs often use various types of equipment and gadgets to help them exercise. Wearable devices measuring some physical or physiological quantity can enhance the process of sensing and provide better coaching advice. The application of science and technology may offer a significant competitive advantage [1]. Wearable technology holds immense potential in the near future for being able to flatten the cost curve of healthcare [2].

Indoor rowing activates multiple muscles and body parts, especially arms (biceps), legs (quadriceps), and stomach muscles. Usually, the indoor rowing machine is equipped with an air resistance mechanism and a sensor that recalculates an applied pulling force. The performance is measured according to this force in each stroke. This device and its internal sensors can be used as research parameters on exercising behaviors [3,4].

In this research, the muscle activation time is measured and processed. Muscle activation time is frequently used to determine the correctness of performed tasks or to identify limits [5,6,7]. Muscle activation can be used to determine muscle fatigue [8,9,10] or can be an indicator of an illness for diagnostic purpose [11,12,13]. Usually, this is performed using surface electromyography (sEMG), but recent studies show the possibility of utilizing other sensors, such as an inertial measurement unit (IMU) [14,15,16], or contactless options with use of motion capture, computer vision, and deep learning pose estimation [17,18,19].

sEMG is capable of detecting muscle contraction time and strength for diagnosis and rehabilitation purposes [20,21]. Despite medical purposes and rehabilitation, sEMG is used to investigate novel approaches to human–robot interaction (HRI). Detecting muscle contractions with sEMG can be used for hand gesture recognition [22,23] or for direct manipulator control [24,25,26].

### 1.1. Contribution

The contribution of this paper is a method of signal processing of sEMG data acquired from biceps and quadriceps during an indoor rowing exercise. A set of parameters is acquired to distinguish the performance of individuals and find common errors in exercising for coaching purposes. The non-athletes’ parameters clearly show common errors when compared to the professional athletes. Moreover, the presented method can distinguish individuals with sports potential among non-athletes.

### 1.2. Paper Structure

Section 2 describes test subjects and test group divisions, a way of preparation for the exercise, sEMG acquisition, and theoretical background on how to utilize sEMG to obtain relevant parameters for performance evaluation. In Section 3, testing presents the separated muscle activation signals and differences between professionals and amateurs. Section 4 presents an evaluation of the parameters with conclusions on the common mistakes of amateurs and advantages of athletes, supported by a precise investigation of calculated parameters. Section 5 presents the conclusions of the research, pointing out what quantities can be used as a base for training recommendations.

## 2. Materials and Methods

### 2.1. sEMG Signal

The sEMG signal can be considered a non-stationary stochastic signal. Its features depend on the level and duration of contraction, the state of muscles (dynamic or static), as well as their fatigue and the contact between electrodes and the skin. While sEMG can be used in pelvic floor evaluation [27,28,29], the typical clinical approach also utilizes non-EMG methodologies [30]. sEMG has also been applied in rehabilitation robots in therapy [20,31,32,33,34,35]. About 95% of the power spectral density of the surface sEMG signal is accounted for by harmonics up to 400 Hz, and the rest is noise connected to electrodes and the device [36]. According to the Kotelnikov–Shannon sampling theorem, to avoid the aliasing phenomenon, the sampling frequency should be equal to at least double the highest frequency contained in the signal, which is at least 1000 Hz [37]. sEMG signal processing is usually based on band-pass filters. The high-pass filter is responsible for softening artifacts developed in patient movements and the unstable contact between electrodes and the skin. On the other hand, the low-pass filter is useful in the removal of noise from electromagnetic radiation and the other devices [36]. Moreover, there is the often-applied filter (50 Hz or 60 Hz) to remove power line interference. In the literature, there is a variety of different parameters applied in the analysis of the sEMG signal. The main parameters of sEMG are the amplitude and length of contraction as well as propagation velocity [38]. sEMG can reveal the “behavior” (i.e., patterns of activity) of a particular muscle, and it can be used to demonstrate whether a muscle is normal, myopathic, or denervated/reinnervated [39]. The parameters are divided into three categories: time domain, frequency domain, and time–frequency domain. The time domain is the most commonly used parameter because of its simplicity and fast computation, as well as the fact that it is based on signal amplitude. The spectral signal analysis is used to examine muscle contraction and deduce changes in the recruitment of motor units [40]. Representation in time–frequency space enables the localization of signal energy in the time domain as well as the frequency domain, so it allows a more accurate description of the physiological phenomenon [36].

**Figure 1:**
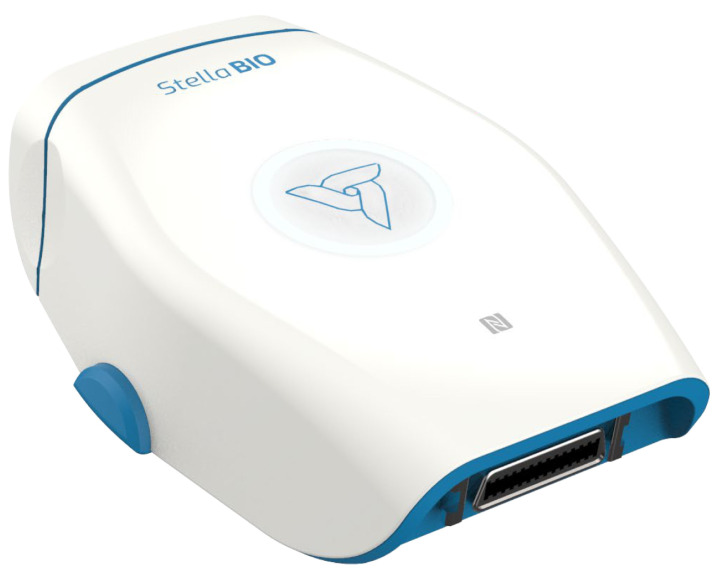
Stella BIO—electromyograph.

**Figure 2:**
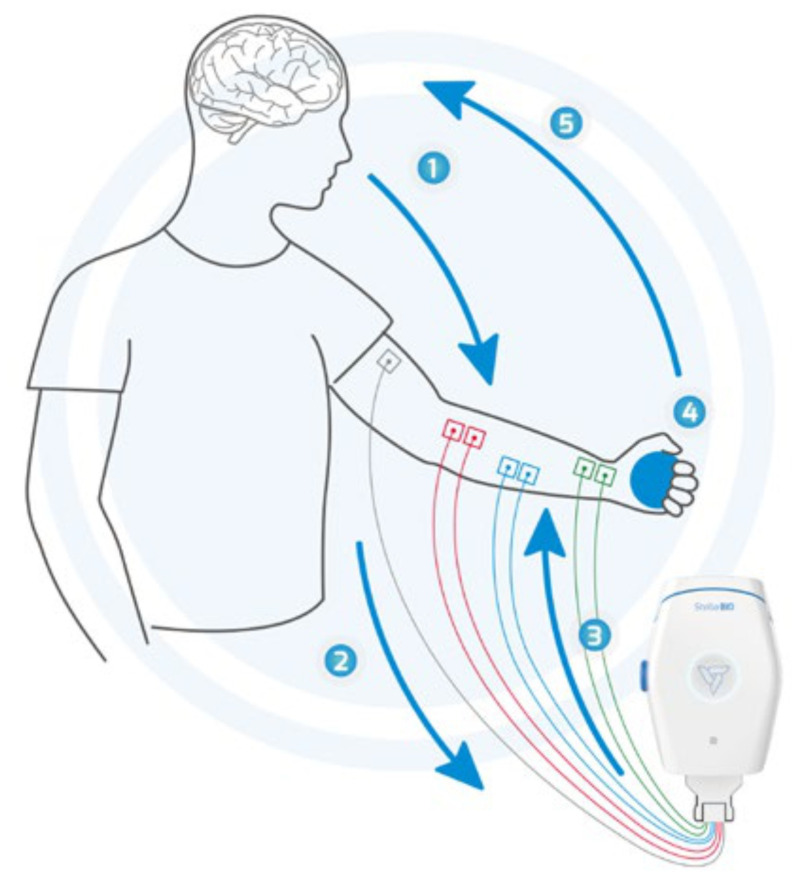
Exemplary Stella BIO electrode connection. (1) Muscle activation, (2) sEMG signal received by Stella BIO, (3) electrostimulation, (4) functional movement, and (5) activity enhancement.

The sEMG signal is gathered with the use of Stella BIO (Figure 1 and Figure 2). Stella BIO is capable of 8-channel sEMG and EMS (electrostimulation) for home and clinical use. In this research, only sEMG as a measurement was used with 3 channels: biceps, quadriceps, and reference.

### 2.2. Training Time

**Figure 3:**
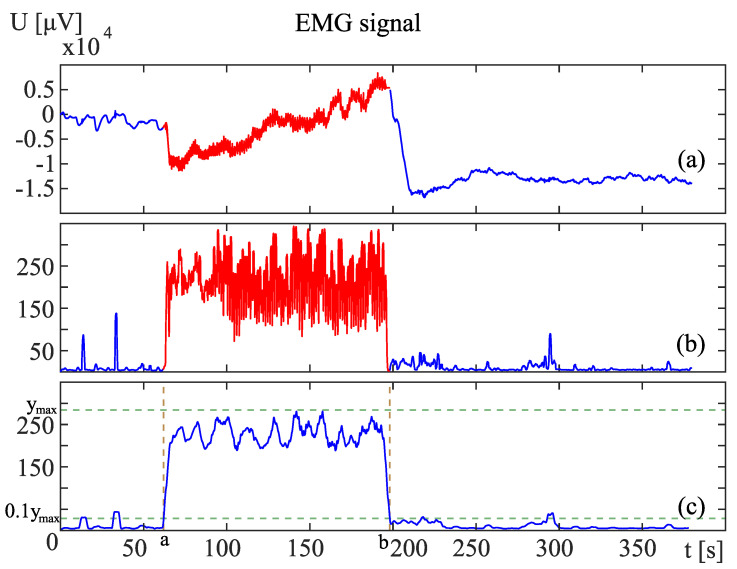
Exemplary sEMG signal recorded during an exercise. The exercise took place from 62 to 198 s, and this region is marked in red. The plots are (**a**) the raw signal, (**b**) the signal transformed with RMS, and (**c**) the filtrated RMS signal.

After the RMS signal is obtained, the next step is to determine the exercise duration. The exercise starts with the first muscle contraction and finishes after the last relaxation. It is characterized by harmonic and rapid variations of the RMS signal in Figure 3b. One can easily observe the exercise in Figure 3b lasts from 62 s to 198 s.

The sEMG signal was collected with the Stella BIO system, with an electromyography accuracy of ±0.5% full scale and baseline noise of <0.5 μV RMS. Stella BIO is a device used both for the evaluation and therapy of neurological, orthopedical, and urogynecology patients. It measures muscle activity using up to 8 channels but also provides electromyography-triggered electrostimulation, functional electrostimulation, and sEMG biofeedback.

The recording starts after all electrodes are attached but before the exercise. Also, after the exercise, the signal is recorded for some time. In this before and after time, some single muscle contractions can occur. Those single spikes can be observed in the example in Figure 3b at 16, 36, and 296 seconds. Moreover, during the exercise, muscle relaxation can bring the signal to values close to zero. These properties do not allow the use of simple thresholds.

To determine the exercise duration automatically, the RMS signal in Figure 3b is filtered to obtain the signal in Figure 3c. The filtration aims to lower the single contractions before and after the exercise and to prevent the signal from droping too low during the experiment. The signal (Figure 3c)

(1) $$y\left(n\right)=\frac{1}{N_{w}}\sum_{i=0}^{N-1}x\left(n-i\right)$$

is obtained by moving averaging filtration with the use of $N_{w}$ samples in a filter window from RMS signal *x*. To smooth the signal, the window size $N_{w}$ is set to be at least equal to $2\hat{t_{c}}\cdot f_{s}$, where $\hat{t_{c}}$ is the average cycle time and $f_{s}$ is the sampling frequency.

The training time,

(2) $$t_{t}=b-a,$$

is the time after exercise start point *a* and before the exercise termination point *b*. Points *a* and *b* are found as the first arguments left and right from the global maximum of the signal *y*, for which the value drops below 0.1 $y_{max}$. The values and formulas provide the proper automatic calculation of start and end points for all recorded exercises.

### 2.3. Training Cycles

The exercise consists of repetitive muscle contractions and relaxations that form a cycle. The signal is split into $N_{c}$ segments according to local minima referring to the moment of muscle being most relaxed. Properly divided signals should have all of the cycle time $t_{c}\approx \hat{t_{c}}$ with a small standard deviation $\sigma_{tc}$. The values $t_{c}\approx 2\hat{t_{c}},3\hat{t_{c}},0.5\hat{t_{c}},\cdots$ usually are the result of wrong minima location and signal split.

**Figure 4:**
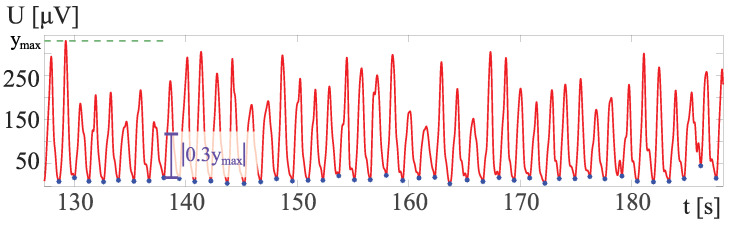
Part of the exemplary sEMG RMS signal from Figure 3b with local minima markings that divide the signal into cycles. The minimum prominence is illustrated with a purple line segment of length 0.3 $y_{max}$.

To find the proper local minima, the minimal prominence is taken into account. The prominence of a trough measures how much the trough stands out because of its intrinsic depth and its location relative to other troughs. During the experiments, the value 0.3 provides the smallest acceptable length of signal segments. Each minimum is marked when the surrounding maxima are over the value of 0.3 $y_{max}$. The part of the signal from Figure 3c with visible cycles, detected local minima, and the visualization of calculated minimal prominence are presented in Figure 4.

The sum of all $N_{c}$ segments time $t_{c}$,

(3) $$\sum_{i=1}^{N_{c}}t_{c}^{\left[i\right]}=t_{t},$$

is the training time $t_{t}$. After the sEMG signal processing, the result that is evaluated is an array of $N_{c}$ signal segments, where each *i* segment shows the muscle activation in one exercise repetition that lasted $t_{c}^{\left[i\right]}$.

### 2.4. Physical Exercise

This research aimed to compare exercise habits between athletes and non-athletes during indoor rowing. The test group contained 13 people, of which 5 were dealing with sports professionally and 8 were sports amateurs. Both groups were comparable in age. The group of athletes was represented by 5 males aged 28–35 years. Members of the non-athletes group were chosen to reflect the athletes’ group in terms of gender and age. All participants were healthy during the experiments. During the exercise, muscle contractions were recorded using Stella BIO with electrodes attached to the biceps and quadriceps.

Each individual was asked to perform an exercise of overcoming 500 m during air-resistance indoor rowing. Properly performed exercise contains several cycles with 4 phases that are as follows:

1. Catch—The initial position with the body ready to start.
2. Drive—The phase of straightening legs and keeping the arms straight. In this phase, the quadricep muscles of the thigh are contracting.
3. Finish—The phase of pulling the bar to the body, contracting the bicep muscles, and leaning back.
4. Recovery—Relaxing muscles and heading back to the catch position.

The experiments were conducted using a Concept 2 rowing machine with default settings. In air-resistance indoor rowing, drag increases with the speed of rowing. This is a classic property used in many devices of this type, so the experiment can be repeated using different machines. The force applied during each cycle is recalculated by the indoor rowing machine to estimate the overcome distance. The exercise finishes after a distance of 500 m is achieved.

## 3. Testing

**Figure 5:**
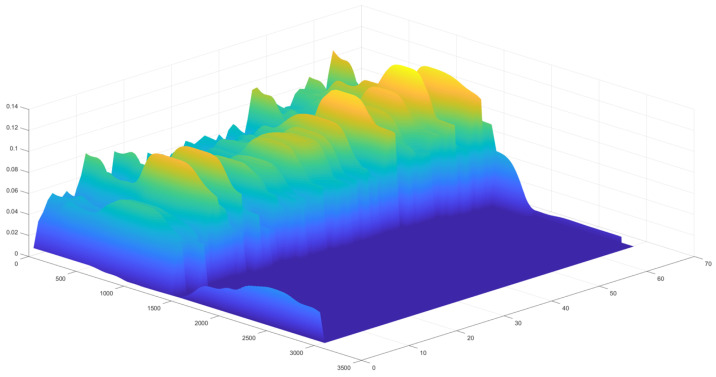
Separation of $N_{c}$ exercise cycles from y(n).

The result of sEMG signal processing, presented in Section 2.1, is the array of $N_{c}$ signal segments $y_{c}^{\left[i\right]}\left(n\right)$ for each muscle. The signal segments are presented in Figure 5. The number of cycles $N_{c}$ and their time $t_{c}^{\left[i\right]}$ vary among individuals. As the sEMG measurement depends on many factors, the values are only comparable among one individual but are not comparable between individuals; thus, the combined plots are normalized.

**Figure 6:**
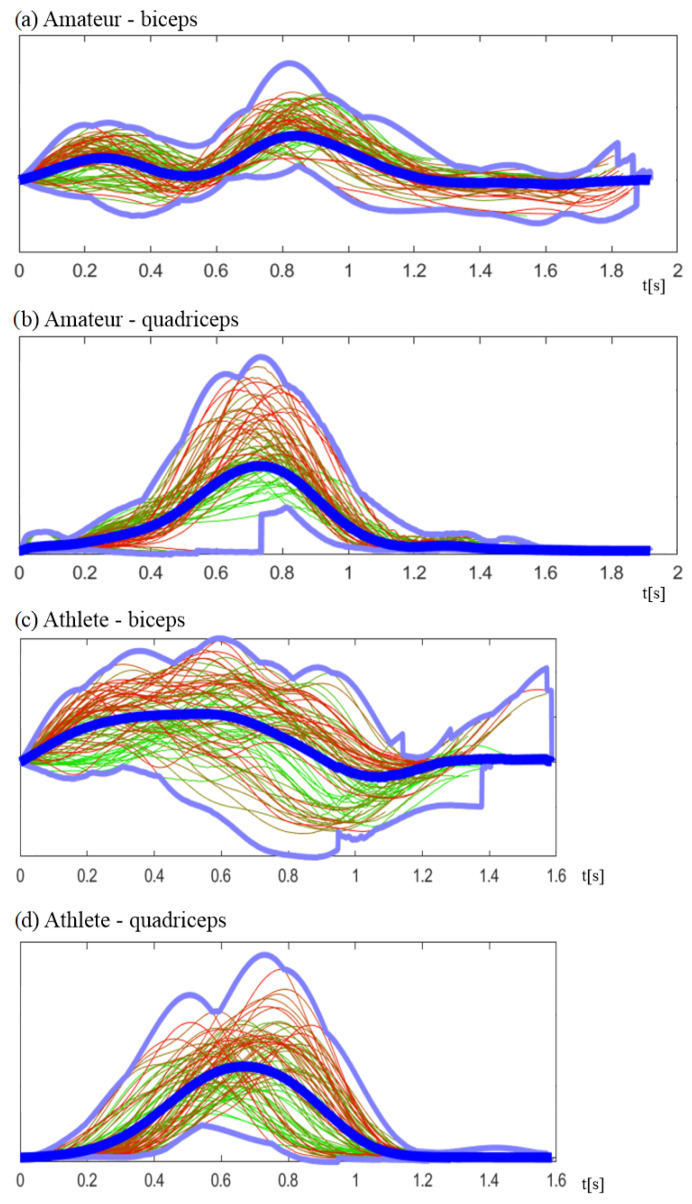
Plots of combined $N_{c}$ exercise cycles $y_{c}^{\left[i\right]}\left(n\right)$ that can be distinguished by the color gradient, where the first cycle is represented in green and the last in red. (**a**,**b**) Amateur and (**c**,**d**) professional sportsman. (**a**,**c**) Quadriceps and (**b**,**d**) biceps. Blue lines are the minimum, maximum, and average muscle activation. Values are normalized because they are not comparable among individuals.

In the properly performed exercise, each phase should be performed in order.This means that the biceps should be activated after the quadriceps. The average plot for professional sportspersons (Figure 6c,d) illustrates the proper exercise performance. After the starting position from phase 1, in t=0.2−0.8 seconds, the quadriceps are activated (phase 2). During the leg straightening, in t=0.2−0.9, the biceps are activated (phase 3). During recovery at t>1.0 (phase 4), the muscles are not active. In the example in Figure 6, an amateur activated the biceps too soon, starting phase 3 before ending phase 2. The local maximum of plot Figure 6a is before that in Figure 6b. Moreover, this mistake caused the need for a second biceps activation at t=0.8 and a second local maximum. However, in this case, the finish phase was shorter and with less efficiency. Thus, one of the variables to determine the correctness of the exercise is the number of local maxima along with the corresponding positions.

In conclusion, based on Equation (3), each exercise can be represented by the number of cycles $N_{c}$, where each cycle has its individual duration time $t_{c}^{\left[i\right]}$. The sum of all $t_{c}^{\left[i\right]}$ in $N_{c}$ is the exercise total time. While an individual can perform shorter cycles or longer cycles, an individual’s physical fitness directly affects the total time $t_{t}$.

## 4. Evaluation

The exercise ends with the array of $N_{c}$ signal segments, and the evaluation process aims to score the individual quality of training. The gathered and processed data are presented in Table 1.

For the eight non-athletes and five athletes, the exercise was evaluated, given the knowledge about six key factors, which are presented in Table 1. Values are conditionally colored: worst values in red, and best are in green. The total exercise time $t_{t}$ in seconds is calculated from Formula (3). The exercise should end as soon as possible. The number of cycle segments $N_{c}$ is the number of performed exercise phases. With a smaller number of cycles, the muscle energy needs to be higher to overcome the set distance; thus, finishing the exercise with a smaller number of cycles can indicate higher muscle power, but this is not a key factor. The standard deviation σ of $t_{c}^{\left[i\right]}$ corresponds to similarities in segment length in seconds. A higher value of σ indicates that the individual had a harder time in keeping the desired rhythm of cycles. The average cycle time $\bar{t^{\left[i\right]}}$ is the average time of the complete exercise phase. Observations about average cycle time are similar to those about the number of cycle segments. Cycle time varies during the exercise. In the evaluation process, the slope of these changes is calculated as $\hat{a}$, which is the slope of the regression line of changing cycle time. The ability to keep the constant cycle rhythm is indicated with a small value of parameter $\hat{a}$. During exercise, there is a point at which an individual is tired and performs the final cycles in a fatigued state. The start of the fatigue phase is calculated as the point at which the approximate muscle energy regression polynomial reaches the maximal value. In Table 1, fatigue indicates how much of the exercise in percentage was performed in the fatigue state.

Parameters 1–4 are directly calculated from the array of signal segments. Parameters $\hat{a}$ and fatigue are explained in Section 4.2.

**Figure 7:**
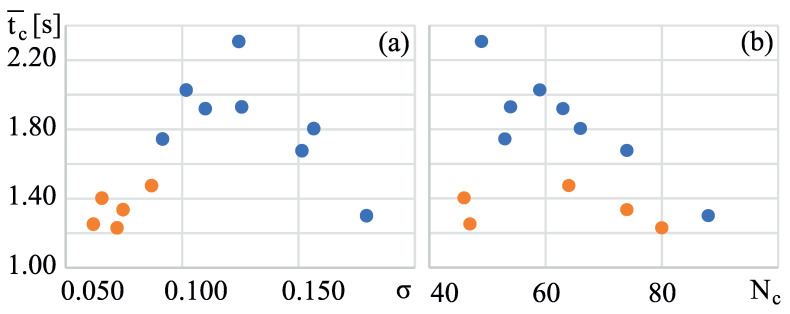
Plots representing data from Table 1 for athletes (orange) and non-athletes (blue). Average cycle time $\bar{t^{\left[i\right]}}$ versus (**a**) standard deviation σ and (**b**) number of cycles $N_{c}$.

Moreover, the data from Table 1 are used to calculate statistical significance (Table 2). For each variable in Table 1 and for each group, the mean value and standard deviation (SD) are calculated to obtain the *t* value and *p* value according to an unpaired *t*-test. As a result, one can observe that by conventional criteria, nearly all suggested variables provide a difference that is considered to be very statistically significant. The only exception is $N_{c}$, which is not statistically significant. This can also be observed in Figure 7b, where $N_{c}$ alone cannot be a classifier.

### 4.1. Cycles and Their Duration

The basic parameters of the exercises are the total exercise time $t_{t}$ and the number of cycles $N_{c}$. Each cycle has its duration; thus, the additional parameters are average cycle time $\bar{t^{\left[i\right]}}$ and its standard deviation σ. The plots of the dependencies of the parameters are presented in Figure 7. The plots show a clear boundary between athletes and non-athletes, and this data can be used to construct a well-performing classifier.

### 4.2. Endurance, Fatigue and Muscle Power

A characteristic feature of athletes is the ability to perform the exercise in a constant rhythm. The cycle time is not so important as long as the individual can maintain it at a constant rate. There are two parameters to measure this ability. The first is the standard deviation,

(4) $$\sigma =\frac{1}{N_{c}-1}\sum_{i=1}^{N_{c}}{\left(t_{c}^{\left[i\right]}-\bar{t_{c}^{\left[i\right]}}\right)}^{2},$$

and the second is the slope of the regression line,

(5) $$y_{reg}\left(i\right)=\hat{a}i+\hat{b}.$$

The $\hat{a}$ and $\hat{b}$ parameters of regression line $y_{reg}$ are calculated in such way that the sum of errors $\left(|y_{reg}\left(i\right)-t_{c}^{\left[i\right]}|\right)$ in each *i* tends to a minimum.

**Figure 8:**
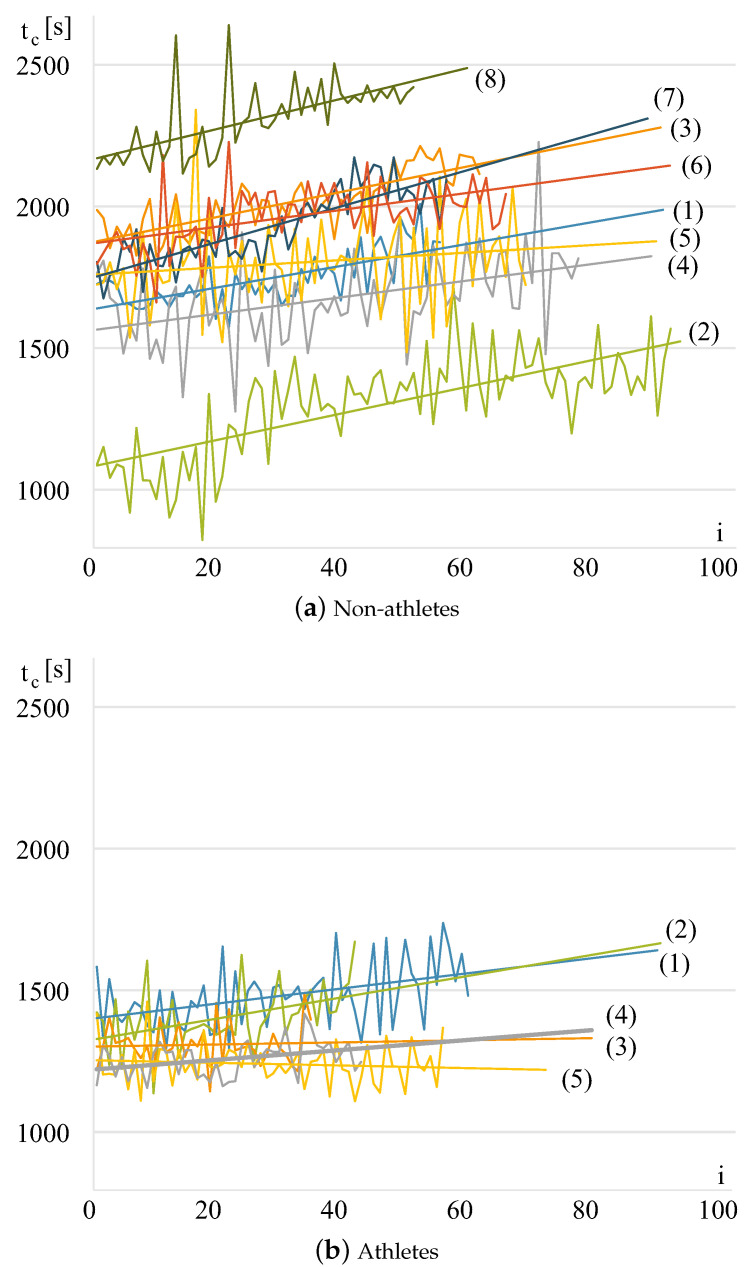
Cycle time $t_{c}^{\left[i\right]}$ changes for individuals from the groups of (**a**) eight non-athletes and (**b**) five athletes. Each plot has a regression line with a slope of $\hat{a}$. Discrete data are drawn as continuous plots to improve the visualization.

As the fatigue grows during exercises, each subsequent cycle requires more time. The more tired a person is, the longer the cycles and the steeper the slope of the regression line $\hat{a}$ (Figure 8). An athlete with good endurance and an ability of strength resource management can plan the amount of applied force; thus, the cycle time will not grow so rapidly.

**Figure 9:**
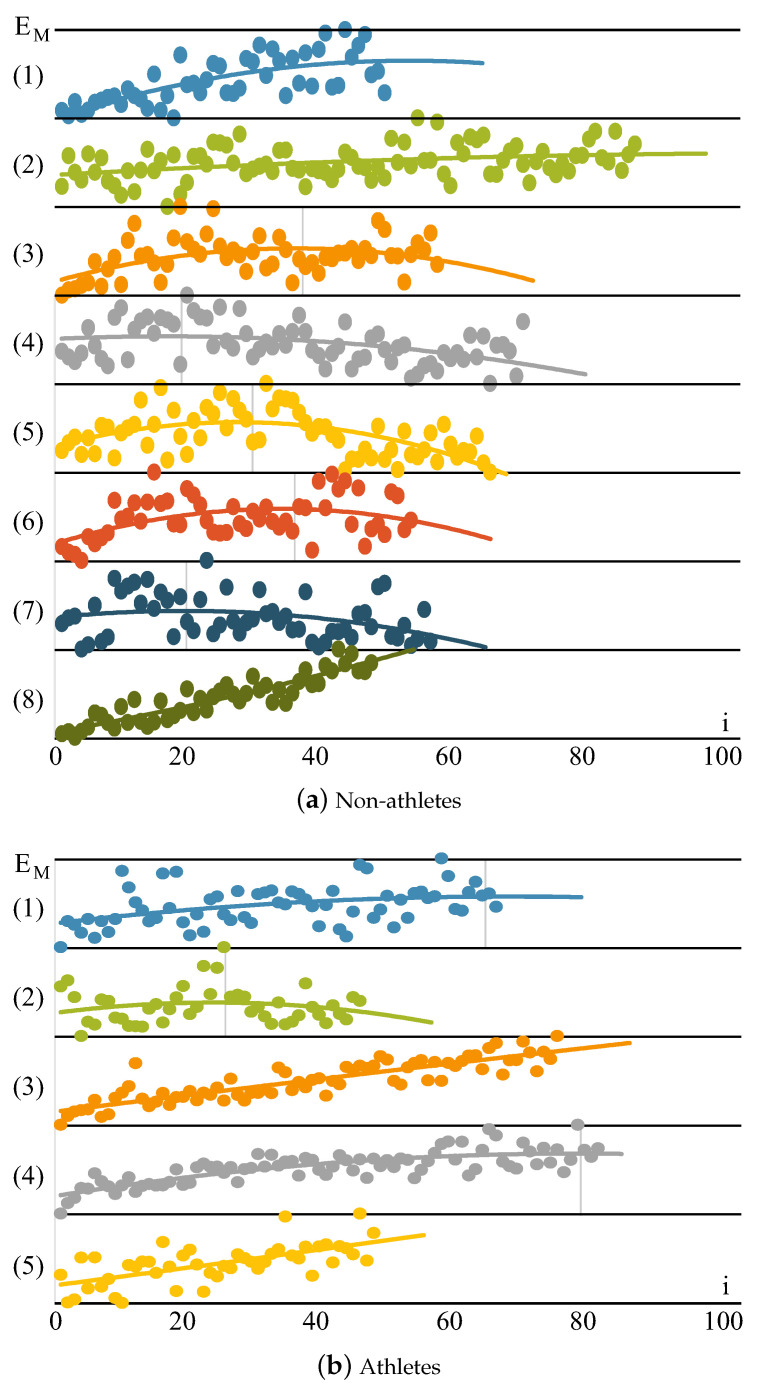
Muscle energy $E_{M}$ change for individuals from the groups of (**a**) eight non-athletes and (**b**) five athletes. $E_{M}$ is not comparable among individuals; thus, the values are normalized. Each plot has a polynomial regression line. The fatigue point is marked as a vertical line.

As the fatigue grows and an individual becomes more tired, more muscle fibers need to be activated. The muscle power, which is the total detected neural force applied on the muscles in each cycle *i*,

(6) $$E_{M}\left[i\right]=\int_{0}^{t_{c}}y_{c}^{\left[i\right]}\left(n\right)\;dn,$$

can be measured as an integral of $y_{c}^{\left[i\right]}\left(n\right)$ signal in each cycle *i* over the $t_{c}$ time. These values are presented in Figure 9.

On the other hand, there is a point at which the individual can no longer activate more muscle fibers. This fatigue point is marked as a vertical line in Figure 9 and can be observed when the muscle power $E_{M}\left[i\right]$ in each subsequent cycle starts to drop. To calculate the fatigue point, first, the polynomial regression line $E_{reg}$ needs to be calculated from $E_{M}\left[i\right]$, and the fatigue point presented in Table 1 is calculated as $t_{t}-i_{max(E_{reg})}/t_{t}\left[\%\right]$, which is the percentage of exercise time that was performed after the maximal muscle power peak. When $t_{t}<i_{max(E_{reg})}$, the fatigue point is equal 0%, as the whole exercise was performed with no extensive fatigue and the individual could do more.

### 4.3. Individual Performance

Based on the data from Table 1, a set of conclusions can be drawn.

The first and most obvious conclusion is that athletes performed the exercise better, more effectively, and with less fatigue. Among the non-athletes, there were some individuals (for example, non-athlete 1) who obtained high scores, probably because of individual training. This is the obvious conclusion, but the contribution of this paper is a mathematical way of proving one fitness, and such conclusions prove the method.

Analyzing second and eighth non-athletes shows that one preferred many short cycles, and the other preferred a small number of longer cycles. Both of them, however, finished the exercises in a similar time and similar $\hat{a}$; thus, this exercise was performed with similar efficiency, proving that $N_{c}$ or $t_{c}$ alone is insignificant.

The non-athletes had a tendency toward increased strength or endurance. Those with high strength finished the exercise faster but could not maintain the constant rhythm and started the fatigue phase early (non-athletes 3, 4, and 6). In this case, the endurance and power management exercise path could be suggested. Those with high endurance (non-athlete 5) performed longer cycles. While they could maintain small $\hat{a}$ or σ, the overall $t_{t}$ was high. In this case, the strength-increasing exercise path could be suggested.

Calculating the fatigue point can indicate when to stop the exercise or if the exercise difficulty should be increased.

### 4.4. Case Study Verification

As a part of the verification of the presented conclusions, a cumulative $y_{c}\left(n\right)$ signal of selected individuals was analyzed closely. Each exercise consisted of $N_{c}$ cycles; thus, this set was split into three equal sets:

- *n* = 0–33% $N_{c}$—warm-up—Early phase, where an individual is at full strength.
- *n* = 33–66% $N_{c}$—exercise—Middle phase, with highest performance.
- *n* = 66–100% $N_{c}$—fatigue—Last phase, where the muscle fatigue is becoming visible.

It is important to notice that the amplitude of the sEMG signal is not a repeatable measurement and is specific to each individual. Therefore, only values within one exercise can be compared. This is the reason why $y_{c}\left(n\right)$ does not show the scale on vertical axes. After comparing plots among one individual, some of the drawn conclusions can be visually verified.

**Figure 10:**
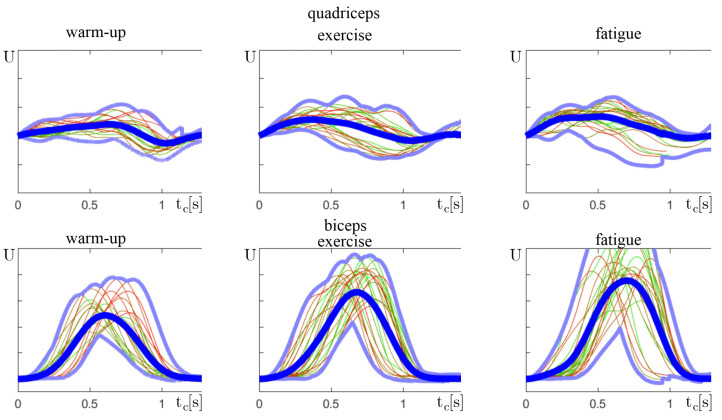
Normalized plots of combined exercise cycles $y_{c}\left(n\right)$, similar to Figure 6, split into three groups.

The first observation states that, as fatigue increases, more muscle fibers are required to be activated to perform the same work. This translates into an increase in the amplitude of the sEMG signal. This can be noticed in Figure 10, especially on the biceps plot, where each phase indicates a higher amplitude. This is an example of an athlete correctly performing the rowing exercise.

**Figure 11:**
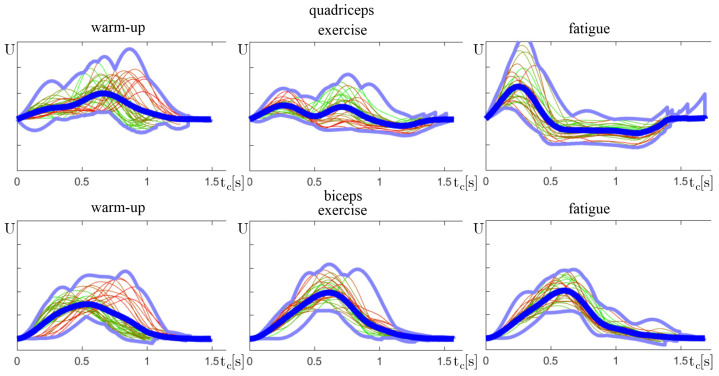
Normalized plots of combined exercise cycles $y_{c}\left(n\right)$, similar to Figure 6, split into three groups.

Some common exercise mistakes can be seen in the cumulative electromyographs of the amateurs. In one case, the exercising person performed too vigorous of movements. They could not keep the rhythm, which is why the graphs do not line up. It is worth observing how the activation time of the leg muscles changed (Figure 11). In the early warm-up phase, the legs were activated after the arms, while in the fatigue phase, because of biceps fatigue, the early rowing movement was performed with the legs first.

**Figure 12:**
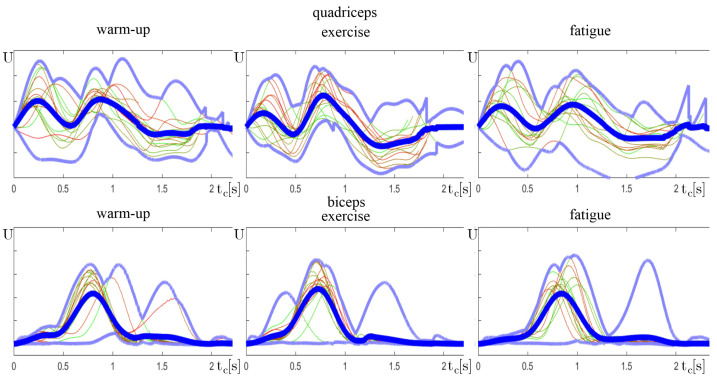
Normalized plots of combined exercise cycles $y_{c}\left(n\right)$, similar to Figure 6, split into three groups.

Keeping the correct exercise rhythm is a key factor in proper exercise performance. Another example (Figure 12) shows that the exercising person was unable to maintain the correct rhythm of the exercise. The repetitions were performed chaotically with a large standard deviation of the cycle length.

## 5. Conclusions

This research proposes a way of measuring individual sports activities. During the experiment, a set of obvious conclusions were obtained: professionals perform the exercise better than amateurs, and fatigue occurs in time during exercise, causing less muscle strength. On the other hand, the most crucial thing is that this research provides a way of measuring these basic phenomena.

Data presented in Table 1 and explained in Section 4 provide a way of measuring one performance based on the muscle activation times and local extrema positions. Section 4.2 shows the way to indicate fatigue, which can be noticed by the changes in cycle amplitude and time, which is an interesting alternative to the median of frequency and its slope during exercise, which is often used during sEMG fatigue assessment [34]. Finally, the exhaustion phase, which can suggest the point of exercise termination, can be noticed as the amplitude of the cycle drops.

Analysis of individual scores presented in Table 1 can bring some valuable comments on one performance that can be a set of recommendations during training.

## Figures and Tables

**Table 1:** Exercise test data. Values for each variable colored according to conditional formatting.

	Individual	$t_{t}\left[s\right]$	$N_{c}$	σ	$\bar{t^{\left[i\right]}}\left[s\right]$	$\hat{a}$	Fatigue
Non-athletes	(1)	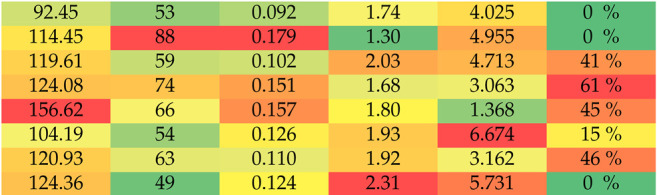					
	(2)						
	(3)						
	(4)						
	(5)						
	(6)						
	(7)						
	(8)						
Athletes	(1)	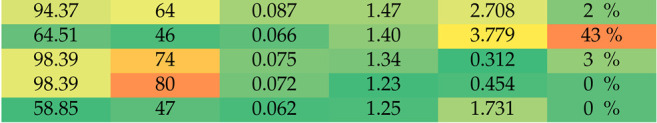					
	(2)						
	(3)						
	(4)						
	(5)						

**Table 2:** Statistical test data.

Variable	Athletes		Non-Athletes		*t* Value	*p* Value
	**Mean**	**SD**	**Mean**	**SD**		
$t_{t}$	119.5863	18.5784	82.9020	19.5450	3.3983	0.0059
$N_{c}$	63.25	12.80	62.20	15.43	0.1333	0.8964
σ	0.13013	0.02988	0.07240	0.00961	4.1275	0.0017
$\bar{t^{\left[i\right]}}$	1.8388	0.2928	1.3380	0.1008	3.6388	0.0039
$\hat{a}$	4.21137	1.67834	1.79680	1.48083	2.6318	0.0233

## Data Availability

Data and materials are not available.
